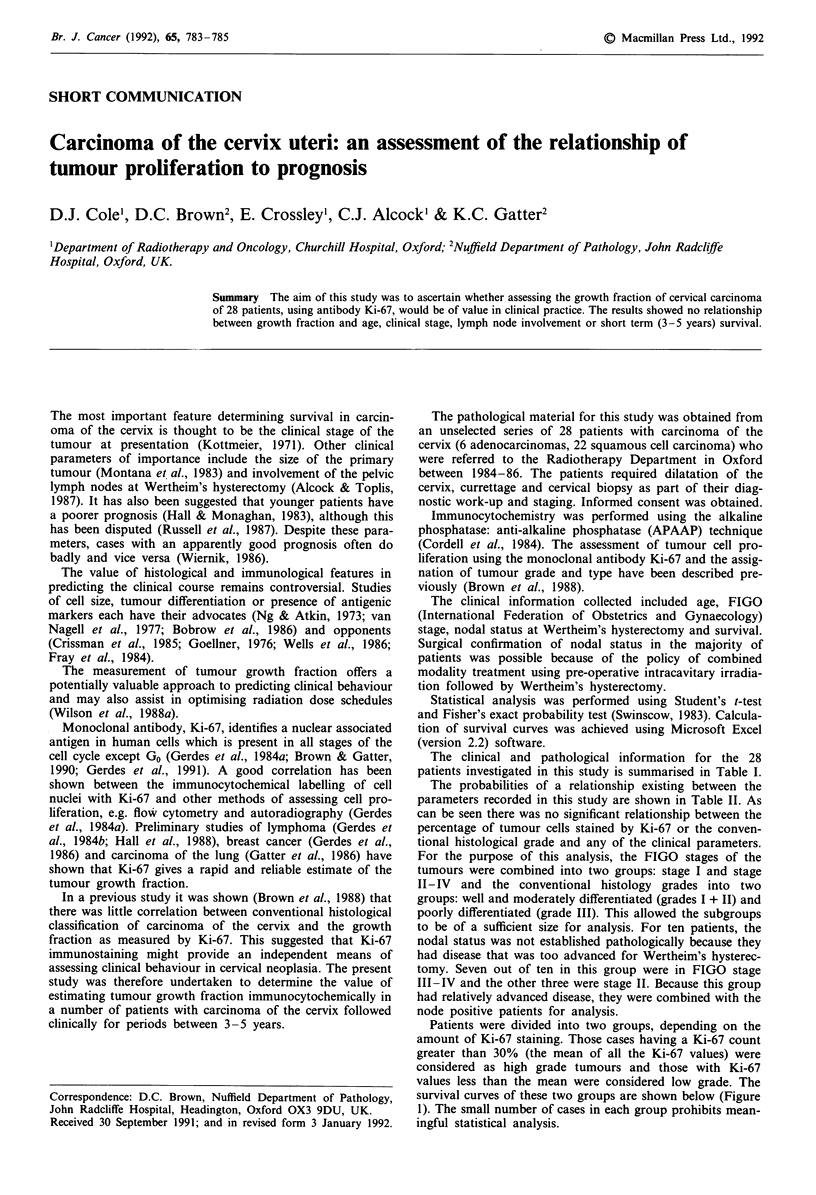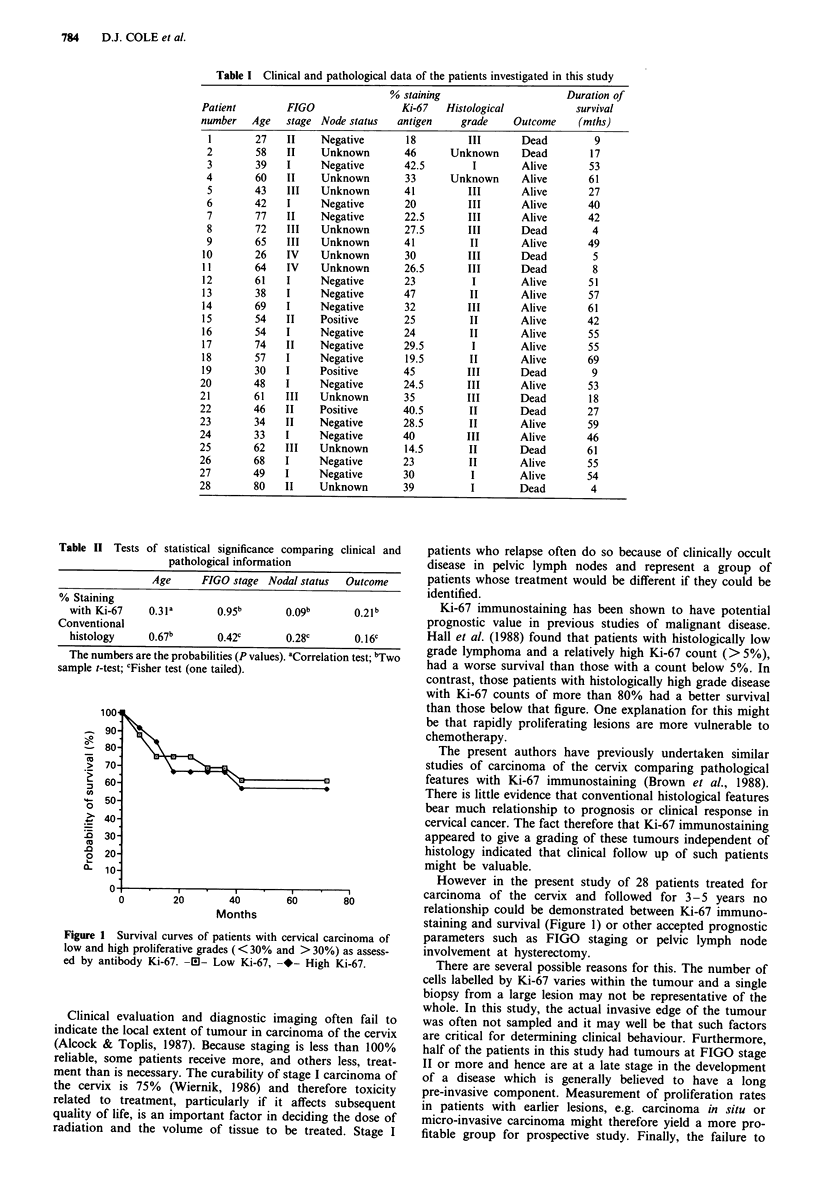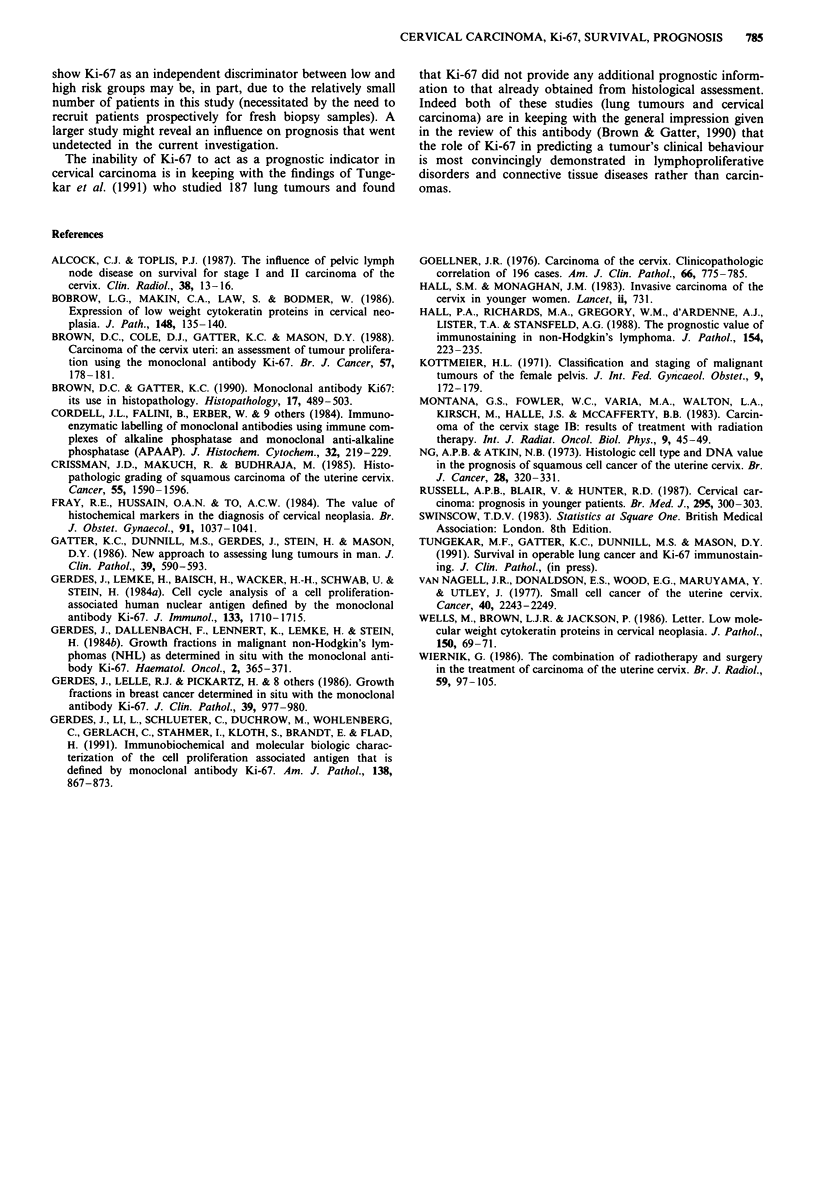# Carcinoma of the cervix uteri: an assessment of the relationship of tumour proliferation to prognosis.

**DOI:** 10.1038/bjc.1992.167

**Published:** 1992-05

**Authors:** D. J. Cole, D. C. Brown, E. Crossley, C. J. Alcock, K. C. Gatter

**Affiliations:** Department of Radiotherapy and Oncology, Churchill Hospital, Oxford, UK.

## Abstract

The aim of this study was to ascertain whether assessing the growth fraction of cervical carcinoma of 28 patients, using antibody Ki-67, would be of value in clinical practice. The results showed no relationship between growth fraction and age, clinical stage, lymph node involvement or short term (3-5 years) survival.


Br. J. Cancer (1992), 65, 783–785 © Macmillan Press Ltd., 1992

SHORT COMMUNICATION

### Carcinoma of the cervix uteri: an assessment of the relationship of tumour proliferation to prognosis

D.J. Cole[1], D.C. Brown[2], E. Crossley[1], C.J. Alcock[1] & K.C. Gatter[2]

[1]*Department of Radiotherapy and Oncology, Churchill Hospital, Oxford;* [2]*Nuffield Department of Pathology, John Radcliffe Hospital, Oxford, UK.*

**Summary** The aim of this study was to ascertain whether assessing the growth fraction of cervical carcinoma of 28 patients, using antibody Ki-67, would be of value in clinical practice. The results showed no relationship between growth fraction and age, clinical stage, lymph node involvement or short term (3–5 years) survival.

The most important feature determining survival in carcinoma of the cervix is thought to be the clinical stage of the tumour at presentation (Kottmeier, 1971). Other clinical parameters of importance include the size of the primary tumour (Montana *et al.*, 1983) and involvement of the pelvic lymph nodes at Wertheim's hysterectomy (Alcock & Toplis, 1987). It has also been suggested that younger patients have a poorer prognosis (Hall & Monaghan, 1983), although this has been disputed (Russell *et al.*, 1987). Despite these parameters, cases with an apparently good prognosis often do badly and vice versa (Wiernik, 1986).

The value of histological and immunological features in predicting the clinical course remains controversial. Studies of cell size, tumour differentiation or presence of antigenic markers each have their advocates (Ng & Atkin, 1973; van Nagell *et al.*, 1977; Bobrow *et al.*, 1986) and opponents (Crissman *et al.*, 1985; Goellner, 1976; Wells *et al.*, 1986; Fray *et al.*, 1984).

The measurement of tumour growth fraction offers a potentially valuable approach to predicting clinical behaviour and may also assist in optimising radiation dose schedules (Wilson *et al.*, 1988*a*).

Monoclonal antibody, Ki-67, identifies a nuclear associated antigen in human cells which is present in all stages of the cell cycle except $G_0$ (Gerdes *et al.*, 1984*a*; Brown & Gatter, 1990; Gerdes *et al.*, 1991). A good correlation has been shown between the immunocytochemical labelling of cell nuclei with Ki-67 and other methods of assessing cell proliferation, e.g. flow cytometry and autoradiography (Gerdes *et al.*, 1984*a*). Preliminary studies of lymphoma (Gerdes *et al.*, 1984*b*; Hall *et al.*, 1988), breast cancer (Gerdes *et al.*, 1986) and carcinoma of the lung (Gatter *et al.*, 1986) have shown that Ki-67 gives a rapid and reliable estimate of the tumour growth fraction.

In a previous study it was shown (Brown *et al.*, 1988) that there was little correlation between conventional histological classification of carcinoma of the cervix and the growth fraction as measured by Ki-67. This suggested that Ki-67 immunostaining might provide an independent means of assessing clinical behaviour in cervical neoplasia. The present study was therefore undertaken to determine the value of estimating tumour growth fraction immunocytochemically in a number of patients with carcinoma of the cervix followed clinically for periods between 3–5 years.

The pathological material for this study was obtained from an unselected series of 28 patients with carcinoma of the cervix (6 adenocarcinomas, 22 squamous cell carcinoma) who were referred to the Radiotherapy Department in Oxford between 1984–86. The patients required dilatation of the cervix, currettage and cervical biopsy as part of their diagnostic work-up and staging. Informed consent was obtained.

Immunocytochemistry was performed using the alkaline phosphatase: anti-alkaline phosphatase (APAAP) technique (Cordell *et al.*, 1984). The assessment of tumour cell proliferation using the monoclonal antibody Ki-67 and the assignation of tumour grade and type have been described previously (Brown *et al.*, 1988).

The clinical information collected included age, FIGO (International Federation of Obstetrics and Gynaecology) stage, nodal status at Wertheim's hysterectomy and survival. Surgical confirmation of nodal status in the majority of patients was possible because of the policy of combined modality treatment using pre-operative intracavitary irradiation followed by Wertheim's hysterectomy.

Statistical analysis was performed using Student's *t*-test and Fisher's exact probability test (Swinscow, 1983). Calculation of survival curves was achieved using Microsoft Excel (version 2.2) software.

The clinical and pathological information for the 28 patients investigated in this study is summarised in Table I.

The probabilities of a relationship existing between the parameters recorded in this study are shown in Table II. As can be seen there was no significant relationship between the percentage of tumour cells stained by Ki-67 or the conventional histological grade and any of the clinical parameters. For the purpose of this analysis, the FIGO stages of the tumours were combined into two groups: stage I and stage II–IV and the conventional histology grades into two groups: well and moderately differentiated (grades I + II) and poorly differentiated (grade III). This allowed the subgroups to be of a sufficient size for analysis. For ten patients, the nodal status was not established pathologically because they had disease that was too advanced for Wertheim's hysterectomy. Seven out of ten in this group were in FIGO stage III–IV and the other three were stage II. Because this group had relatively advanced disease, they were combined with the node positive patients for analysis.

Patients were divided into two groups, depending on the amount of Ki-67 staining. Those cases having a Ki-67 count greater than 30% (the mean of all the Ki-67 values) were considered as high grade tumours and those with Ki-67 values less than the mean were considered low grade. The survival curves of these two groups are shown below (Figure 1). The small number of cases in each group prohibits meaningful statistical analysis.

Correspondence: D.C. Brown, Nuffield Department of Pathology, John Radcliffe Hospital, Headington, Oxford OX3 9DU, UK.
Received 30 September 1991; and in revised form 3 January 1992.

Table I Clinical and pathological data of the patients investigated in this study

| Patient number | Age | FIGO stage | Node status | % staining Ki-67 antigen | Histological grade | Outcome | Duration of survival (mths) |
|---|---|---|---|---|---|---|---|
| 1 | 27 | II | Negative | 18 | III | Dead | 9 |
| 2 | 58 | II | Unknown | 46 | Unknown | Dead | 17 |
| 3 | 39 | I | Negative | 42.5 | I | Alive | 53 |
| 4 | 60 | II | Unknown | 33 | Unknown | Alive | 61 |
| 5 | 43 | III | Unknown | 41 | III | Alive | 27 |
| 6 | 42 | I | Negative | 20 | III | Alive | 40 |
| 7 | 77 | II | Negative | 22.5 | III | Alive | 42 |
| 8 | 72 | III | Unknown | 27.5 | III | Dead | 4 |
| 9 | 65 | III | Unknown | 41 | II | Alive | 49 |
| 10 | 26 | IV | Unknown | 30 | III | Dead | 5 |
| 11 | 64 | IV | Unknown | 26.5 | III | Dead | 8 |
| 12 | 61 | I | Negative | 23 | I | Alive | 51 |
| 13 | 38 | I | Negative | 47 | II | Alive | 57 |
| 14 | 69 | I | Negative | 32 | III | Alive | 61 |
| 15 | 54 | II | Positive | 25 | II | Alive | 42 |
| 16 | 54 | I | Negative | 24 | II | Alive | 55 |
| 17 | 74 | II | Negative | 29.5 | I | Alive | 55 |
| 18 | 57 | I | Negative | 19.5 | II | Alive | 69 |
| 19 | 30 | I | Positive | 45 | III | Dead | 9 |
| 20 | 48 | I | Negative | 24.5 | III | Alive | 53 |
| 21 | 61 | III | Unknown | 35 | III | Dead | 18 |
| 22 | 46 | II | Positive | 40.5 | II | Dead | 27 |
| 23 | 34 | II | Negative | 28.5 | II | Alive | 59 |
| 24 | 33 | I | Negative | 40 | III | Alive | 46 |
| 25 | 62 | III | Unknown | 14.5 | II | Dead | 61 |
| 26 | 68 | I | Negative | 23 | II | Alive | 55 |
| 27 | 49 | I | Negative | 30 | I | Alive | 54 |
| 28 | 80 | II | Unknown | 39 | I | Dead | 4 |

Table II Tests of statistical significance comparing clinical and pathological information

| | Age | FIGO stage | Nodal status | Outcome |
|---|---|---|---|---|
| % Staining with Ki-67 | 0.31[a] | 0.95[b] | 0.09[b] | 0.21[b] |
| Conventional histology | 0.67[b] | 0.42[c] | 0.28[c] | 0.16[c] |

The numbers are the probabilities (P values). [a]Correlation test; [b]Two sample t-test; [c]Fisher test (one tailed).

Figure 1 Survival curves of patients with cervical carcinoma of low and high proliferative grades (<30% and >30%) as assessed by antibody Ki-67. -◻- Low Ki-67, -◆- High Ki-67.

Clinical evaluation and diagnostic imaging often fail to indicate the local extent of tumour in carcinoma of the cervix (Alcock & Toplis, 1987). Because staging is less than 100% reliable, some patients receive more, and others less, treatment than is necessary. The curability of stage I carcinoma of the cervix is 75% (Wiernik, 1986) and therefore toxicity related to treatment, particularly if it affects subsequent quality of life, is an important factor in deciding the dose of radiation and the volume of tissue to be treated. Stage I patients who relapse often do so because of clinically occult disease in pelvic lymph nodes and represent a group of patients whose treatment would be different if they could be identified.

Ki-67 immunostaining has been shown to have potential prognostic value in previous studies of malignant disease. Hall et al. (1988) found that patients with histologically low grade lymphoma and a relatively high Ki-67 count (>5%), had a worse survival than those with a count below 5%. In contrast, those patients with histologically high grade disease with Ki-67 counts of more than 80% had a better survival than those below that figure. One explanation for this might be that rapidly proliferating lesions are more vulnerable to chemotherapy.

The present authors have previously undertaken similar studies of carcinoma of the cervix comparing pathological features with Ki-67 immunostaining (Brown et al., 1988). There is little evidence that conventional histological features bear much relationship to prognosis or clinical response in cervical cancer. The fact therefore that Ki-67 immunostaining appeared to give a grading of these tumours independent of histology indicated that clinical follow up of such patients might be valuable.

However in the present study of 28 patients treated for carcinoma of the cervix and followed for 3–5 years no relationship could be demonstrated between Ki-67 immunostaining and survival (Figure 1) or other accepted prognostic parameters such as FIGO staging or pelvic lymph node involvement at hysterectomy.

There are several possible reasons for this. The number of cells labelled by Ki-67 varies within the tumour and a single biopsy from a large lesion may not be representative of the whole. In this study, the actual invasive edge of the tumour was often not sampled and it may well be that such factors are critical for determining clinical behaviour. Furthermore, half of the patients in this study had tumours at FIGO stage II or more and hence are at a late stage in the development of a disease which is generally believed to have a long pre-invasive component. Measurement of proliferation rates in patients with earlier lesions, e.g. carcinoma *in situ* or micro-invasive carcinoma might therefore yield a more profitable group for prospective study. Finally, the failure to show Ki-67 as an independent discriminator between low and high risk groups may be, in part, due to the relatively small number of patients in this study (necessitated by the need to recruit patients prospectively for fresh biopsy samples). A larger study might reveal an influence on prognosis that went undetected in the current investigation.

The inability of Ki-67 to act as a prognostic indicator in cervical carcinoma is in keeping with the findings of Tungekar *et al.* (1991) who studied 187 lung tumours and found that Ki-67 did not provide any additional prognostic information to that already obtained from histological assessment. Indeed both of these studies (lung tumours and cervical carcinoma) are in keeping with the general impression given in the review of this antibody (Brown & Gatter, 1990) that the role of Ki-67 in predicting a tumour's clinical behaviour is most convincingly demonstrated in lymphoproliferative disorders and connective tissue diseases rather than carcinomas.

### References

ALCOCK, C.J. & TOPLIS, P.J. (1987). The influence of pelvic lymph node disease on survival for stage I and II carcinoma of the cervix. *Clin. Radiol.*, **38**, 13-16.

BOBROW, L.G., MAKIN, C.A., LAW, S. & BODMER, W. (1986). Expression of low weight cytokeratin proteins in cervical neoplasia. *J. Path.*, **148**, 135-140.

BROWN, D.C., COLE, D.J., GATTER, K.C. & MASON, D.Y. (1988). Carcinoma of the cervix uteri: an assessment of tumour proliferation using the monoclonal antibody Ki-67. *Br. J. Cancer*, **57**, 178-181.

BROWN, D.C. & GATTER, K.C. (1990). Monoclonal antibody Ki67: its use in histopathology. *Histopathology*, **17**, 489-503.

CORDELL, J.L., FALINI, B., ERBER, W. & 9 others (1984). Immunoenzymatic labelling of monoclonal antibodies using immune complexes of alkaline phosphatase and monoclonal anti-alkaline phosphatase (APAAP). *J. Histochem. Cytochem.*, **32**, 219-229.

CRISSMAN, J.D., MAKUCH, R. & BUDHRAJA, M. (1985). Histopathologic grading of squamous carcinoma of the uterine cervix. *Cancer*, **55**, 1590-1596.

FRAY, R.E., HUSSAIN, O.A.N. & TO, A.C.W. (1984). The value of histochemical markers in the diagnosis of cervical neoplasia. *Br. J. Obstet. Gynaecol.*, **91**, 1037-1041.

GATTER, K.C., DUNNILL, M.S., GERDES, J., STEIN, H. & MASON, D.Y. (1986). New approach to assessing lung tumours in man. *J. Clin. Pathol.*, **39**, 590-593.

GERDES, J., LEMKE, H., BAISCH, H., WACKER, H.-H., SCHWAB, U. & STEIN, H. (1984*a*). Cell cycle analysis of a cell proliferation-associated human nuclear antigen defined by the monoclonal antibody Ki-67. *J. Immunol.*, **133**, 1710-1715.

GERDES, J., DALLENBACH, F., LENNERT, K., LEMKE, H. & STEIN, H. (1984*b*). Growth fractions in malignant non-Hodgkin's lymphomas (NHL) as determined in situ with the monoclonal antibody Ki-67. *Haematol. Oncol.*, **2**, 365-371.

GERDES, J., LELLE, R.J. & PICKARTZ, H. & 8 others (1986). Growth fractions in breast cancer determined in situ with the monoclonal antibody Ki-67. *J. Clin. Pathol.*, **39**, 977-980.

GERDES, J., LI, L., SCHLUETER, C., DUCHROW, M., WOHLENBERG, C., GERLACH, C., STAHMER, I., KLOTH, S., BRANDT, E. & FLAD, H. (1991). Immunobiochemical and molecular biologic characterization of the cell proliferation associated antigen that is defined by monoclonal antibody Ki-67. *Am. J. Pathol.*, **138**, 867-873.

GOELLNER, J.R. (1976). Carcinoma of the cervix. Clinicopathologic correlation of 196 cases. *Am. J. Clin. Pathol.*, **66**, 775-785.

HALL, S.M. & MONAGHAN, J.M. (1983). Invasive carcinoma of the cervix in younger women. *Lancet*, **ii**, 731.

HALL, P.A., RICHARDS, M.A., GREGORY, W.M., d'ARDENNE, A.J., LISTER, T.A. & STANSFELD, A.G. (1988). The prognostic value of immunostaining in non-Hodgkin's lymphoma. *J. Pathol.*, **154**, 223-235.

KOTTMEIER, H.L. (1971). Classification and staging of malignant tumours of the female pelvis. *J. Int. Fed. Gyncaeol. Obstet.*, **9**, 172-179.

MONTANA, G.S., FOWLER, W.C., VARIA, M.A., WALTON, L.A., KIRSCH, M., HALLE, J.S. & MCCAFFERTY, B.B. (1983). Carcinoma of the cervix stage IB: results of treatment with radiation therapy. *Int. J. Radiat. Oncol. Biol. Phys.*, **9**, 45-49.

NG, A.P.B. & ATKIN, N.B. (1973). Histologic cell type and DNA value in the prognosis of squamous cell cancer of the uterine cervix. *Br. J. Cancer*, **28**, 320-331.

RUSSELL, A.P.B., BLAIR, V. & HUNTER, R.D. (1987). Cervical carcinoma: prognosis in younger patients. *Br. Med. J.*, **295**, 300-303.

SWINSCOW, T.D.V. (1983). *Statistics at Square One*. British Medical Association: London. 8th Edition.

TUNGEKAR, M.F., GATTER, K.C., DUNNILL, M.S. & MASON, D.Y. (1991). Survival in operable lung cancer and Ki-67 immunostaining. *J. Clin. Pathol.*, (in press).

VAN NAGELL, J.R., DONALDSON, E.S., WOOD, E.G., MARUYAMA, Y. & UTLEY, J. (1977). Small cell cancer of the uterine cervix. *Cancer*, **40**, 2243-2249.

WELLS, M., BROWN, L.J.R. & JACKSON, P. (1986). Letter. Low molecular weight cytokeratin proteins in cervical neoplasia. *J. Pathol.*, **150**, 69-71.

WIERNIK, G. (1986). The combination of radiotherapy and surgery in the treatment of carcinoma of the uterine cervix. *Br. J. Radiol.*, **59**, 97-105.